# Reasons for (Non)Participating in a Telephone-Based Intervention Program for Families with Overweight Children

**DOI:** 10.1371/journal.pone.0034580

**Published:** 2012-04-03

**Authors:** Franziska Alff, Jana Markert, Silke Zschaler, Ruth Gausche, Wieland Kiess, Susann Blüher

**Affiliations:** 1 Department of Women and Child Health, Hospital for Children and Adolescents, University Hospital of Leipzig, Leipzig, Germany; 2 Integrated Research and Treatment Center (IFB) AdiposityDiseases, University of Leipzig, Leipzig, Germany; 3 CrescNet gGmbH, University of Leipzig, Leipzig, Germany; Louisiana State University, Pennington Biomedical Research Center, United States of America

## Abstract

**Objective:**

Willingness to participate in obesity prevention programs is low; underlying reasons are poorly understood. We evaluated reasons for (non)participating in a novel telephone-based obesity prevention program for overweight children and their families.

**Method:**

Overweight children and adolescents (BMI>90^th^ percentile) aged 3.5–17.4 years were screened via the CrescNet database, a representative cohort of German children, and program participation (repetitive computer aided telephone counseling) was offered by their local pediatrician. Identical questionnaires to collect baseline data on anthropometrics, lifestyle, eating habits, sociodemographic and psychosocial parameters were analyzed from 433 families (241 participants, 192 nonparticipants). Univariate analyses and binary logistic regression were used to identify factors associated with nonparticipation.

**Results:**

The number of overweight children (BMI>90^th^ percentile) was higher in nonparticipants than participants (62% vs. 41.1%,p<0.001), whereas the number of obese children (BMI>97^th^ percentile) was higher in participants (58.9% vs.38%,p<0.001). Participating girls were younger than boys (8.8 vs.10.4 years, p<0.001). 87.3% and 40% of participants, but only 72.2% and 24.7% of nonparticipants, respectively, reported to have regular breakfasts (p = 0.008) and 5 regular daily meals (p = 0.003). Nonparticipants had a lower household-net-income (p<0.001), but higher subjective physical wellbeing than participants (p = 0.018) and believed that changes in lifestyle can be made easily (p = 0.05).

**Conclusion:**

An important reason for nonparticipation was non-awareness of their child's weight status by parents. Nonparticipants, who were often low-income families, believed that they already perform a healthy lifestyle and had a higher subjective wellbeing. We hypothesize that even a low-threshold intervention program does not reach the families who really need it.

## Introduction

In the past decades, the prevalence of childhood obesity has increased dramatically [1], although prevalence rates seem to have stabilized at a high level [2]. Childhood obesity needs to be effectively treated, starting at a young age because its high degree of persistence represents a major risk factor leading to the development of cardiovascular [3] and metabolic diseases [4].

Obesity in childhood is a key predictor for obesity in adulthood [5]. Therefore, children and adolescents should be considered as a priority population for prevention. As obesity tends to affect the whole family, treatment of childhood overweight or obesity should involve the entire family environment [6]–[8]. Family-based programs have also reliably produced the best short- and long-term effects on weight loss of affected children [9]. Therefore, family-based interventions, with the parents as the main agents of change, are highly recommended [10]. However, the participation rates of families in prevention programs are rather low [11]. It is important to identify barriers and incentives leading to or impeding participation in prevention programs. Family participation is a minimum prerequisite for program efficacy [12]. The subjective need for treatment seems to be rather low in most families with overweight children [13] which might be partly attributable to the fact that parents might be not aware of the child's weight status [14]. However, factors that encourage or discourage participation in prevention programs have been widely neglected. In the present study, we have analyzed for the first time in Germany reasons for (non)participation in a novel, low-threshold, telephone-based obesity prevention program for families with overweight children (T.A.F.F.: **T**elephone-based **A**diposity prevention **F**or **F**amilies) in a representative cohort of German children.

## Methods

### Study design

T.A.F.F. (Telephone-based Adiposity Prevention for Families) is a novel low-threshold, telephone based obesity prevention program (randomized clinical trial with an intervention group and a control group without intervention) for families with overweight (BMI>90^th^ percentile according to the German reference values) [15] children and adolescents aged 3.5–17.4 years. Eligible families who denied program participation where asked to complete identical questionnaires than participating families to obtain comparable baseline data on lifestyle, eating habits, sociodemographic, anthropometric, and psychosocial parameters. The study questionnaires addressed mothers, fathers, and children (older than ten years) separately, thus providing comprehensive baseline data of mother-father-child triads. The study design was approved by the local ethics committee of the faculty of medicine, University of Leipzig, and the entire study has been carried out according to the ethical principles originating from the Declaration of Helsinki and are consistent with ICH-GCP guidelines. Written informed consent was obtained from all participants involved in our study.

### Recruitment

Program recruitment was performed via the German CrescNet database, a representative data collection of German children, which monitors actually body weight and body height data from over 550,000 children from all over Germany [16]. Ultimately, 317 pediatricians participate in the computerized data collection, who are supplied with the same type of stadiometer and a device according to anthropometric measuring standards. Body weight is measured using medically calibrated scales with children wearing only light underwear. One of the main objectives of CrescNet is to ensure that abnormal growth and weight development are detected at a very early stage in order to offer the affected families the appropriate treatment. Eligible candidates screened via the CrescNet database according to the inclusion criteria (see below) were pseudonymized communicated to their pediatrician, who informed the family about the child's weight status and offered program participation. Out of the 227 families who denied program participation but sent their written informed consent to complete the questionnaires for evaluation of reasons for nonparticipation, 35 had to be excluded due to non-modifiable reasons for nonparticipation (see below). The baseline data of the remaining 192 families (further referred to as nonparticipants; 107 boys, 85 girls) was compared to that of 241 participating families (further referred to as participants; 117 boys, 124 girls) for analyses of reasons for (non)participation. Complete questionnaires of family triads (mother, father, child>10 years) were obtained from 210 families (110 participants, 100 nonparticipants).

**Inclusion and exclusion criteria:** Children and adolescents aged 3.5–17.4 years, BMI>90th percentile (last measurement within the past 6 months by local pediatrician) were included. Written informed consent to participate in the intervention or in the analysis of reasions for (non)participation had to be returned to the study center.

Exclusion criteria were program drop-out of participants within the first seven ( = one half of intervention) counseling interviews, in order to evaluate only candidates adhering to the offered program, and serious chronic disease underlying the child's overweight.

### Measurements and tools

**Anthropometric parameters:** Body weight and body height were measured by the local pediatrician and transferred to the CrescNet database. Individual BMI was calculated (weight divided by the square of height; kg/m^2^). Patients were classified in three groups according to the German sex- and age-specific reference percentiles: overweight (BMI 90^th^–97^th^ percentile), obese (BMI≥97^th^ percentile) and extremely obese (BMI≥99.5^th^ percentile) [15]. For further analyses, all individual BMI data were also converted into standard deviation scores (BMI-SDS) using the LMS method [17]. BMI values of the parents were based on self-reported heights and weights, gathered from the questionnaires. The classification for parental weight status was BMI = 18.5–24.9 kg/m^2^ for normal weight, BMI>25 kg/m^2^ for overweight and BMI>30 kg/m^2^ for obese individuals [18].

**Tools to assess child, parental, and family characteristics:** *C*hildren (older than 10 years) and their parents (mothers and fathers separately) completed a structured questionnaire asking for lifestyle factors, daily physical activity, leisure time habits, media consumption, psychosocial functioning as well as socio-demographic variables (marital status; parental employment: “no employment”, “working part-time”, “working full-time;” parental level of education: “high” for 12 years of school attendance, “middle” for 10 years of school attendance, “low” for 9 years or less of school attendance; household-net-income: low household-net-income (<2000 €), middle household-net-income (2000–4000€) and high household-net-income (>4000 €); place of residence: village (population<5.000), small town (population 5.000–20.000), mid-size town (population 20.000–100.000), big city (population>100.000)).

**Lifestyle variables:** Parameters to characterize lifestyle included meal frequencies of the child, ranging from 1–5 (early breakfast taken at home, late breakfast taken at school, lunch, afternoon meal, dinner), physical activity ranging from every day to never (3–5 times a week, 1–2 times a week, 1–2 times a month), media consumption and leisure time habits. The information on whether a child was a member of a sports club (yes/no) was also included in the analysis. The items are based on the questionnaires used by the German health Interview and Examination Survey for children and adolescents (KiGGS) [19].

**Behavioral variables:** Stages of behavioral change in lifestyle were determined according to the Health Action Process Approach (HAPA) [20]. The HAPA model identifies specific stages that individuals go through when trying to change their behavior. The model suggests that the adoption, initiation, and maintenance of health behaviors must be explicitly conceived as a process that consists of at least a motivation phase and a volition phase. The latter might be further subdivided into a planning phase, action phase, and maintenance phase. It is claimed that perceived self-efficacy plays a crucial role at all stages, along with other cognitions.

**Health-related quality of life:** Health-related quality of life was assessed by self-report, using the validated and evaluated quality-of-life questionnaire (Kindl-R) [21]. The Kindl-R comprises 24 items, which can be responded to on a 5-point Likert-scale (never, seldom, sometimes, often, all the time). The resulting subscales include the following categories: physical well-being, emotional well-being, self-esteem, family environment, interaction with friends, and everyday functioning. The subscales of these 6 dimensions can be combined to produce a total score. Psychometric results revealed a high degree of reliability (Cronbach's Alpha ± 0.70) and satisfactory convergent validity of the procedure. Age-specific versions consider changes in the quality of life dimensions over the course of the child's development.

### Reasons for nonparticipation

To identify the family's perception of reasons that influenced their program participation the following question was asked: “Why did you decide not to participate in the program?” Multiple “closed” (Family uses other offers, time constraints, parental perception of children's overweight status, parental perception of children's health status, financial arguments [yes, no]) and “open” answers were possible.

### Statistical analysis

Statistical analysis was performed using the SPSS statistical software package version 18.0 (SPSS Inc., Chicago II, USA). Data are presented as mean+-standard deviation (SD) or as mean (M) (percentage in parentheses). Group means were compared using chi-square tests for categorical data and independent t-tests for continuous data. The Mann-Whitney U-test was used for ordinal data (non-parametric) to compare the group means. Within-group differences were tested using a binominal test. Binary logistic regression was used to describe the relationship between variables and program participation. The multivariate model was then constructed with variables that had a univariate p-value of 0.1 or less, and the level of significance was set at α<0.05.

## Results

### Modifiable reasons for nonparticipation

**Baseline anthropometrics in children and adolescents:** The prevalence of overweight was 41.1% in participants and 62.0% in nonparticipants. The prevalence of obesity was 58.9% in participants and 38.0% in nonparticipants (X^2^[2, N = 433] = 19.88, p<0.001) (Table 1).

**Table 1:** Baseline characteristics of participating and nonparticipating children.

Variable	Participants	Nonparticipants	P-value
**Anthropometric measurement**	**(n = 241)**	**(n = 192)**	
Age (mean ±SD)	9.58±3.07	9.68±3.57	P = 0.722
BMI-SDS (mean ±SD)	2.02±0.46	1.84±0.46	P<0.001*
Sex			P = 0.137
*Boys*	117 (52.2%)	107 (47.8%)	
Age (mean ±SD)	10.36±2.97	9.66±3.51	P = 0.092
BMI (mean ±SD)	2.03±0.41	1.87±0.48	P = 0.001*
*Girls*	124 (59.3%)	85 (40.7%)	P = 0.008*
Age (mean ±SD)	8.84±3.0	9.70±3.65	P = 0.106
BMI (mean ±SD)	2.02±0.51	1.80±0.43	P<0.001*
**Meal Pattern**	**(n = 110)**	**(n = 100)**	
Meal frequency			p = 0.003*
*1*	0.9%	1%	
*2*	0.9%	4.1%	
*3*	9.1%	19.6%	
*4*	49.1%	50.5%	
*5*	40.0%	24. 7%	
Breakfast consumption	87.3%	72.2%	P = 0.008*
**Physical Activity**	**(n = 110)**	**(n = 100)**	
Leisure-time physical activity			P = 0.587
*Every day*	30.3%	22.1%	
*3–5 times a week*	27.5%	29.5%	
*1–2 times a week*	31.2%	34.7%	
*1–2 times a month*	6.4%	5.3%	
*Less or never*	4.6%	8.4%	
Sport club membership	57.8%	46.4%	P = 0.102

(Baseline anthropometrics for 241 participants and 192 nonparticipants; additional parameters (completed questionnaires by mother-father-child-triads) for 110 participants and 100 nonparticipants).

Statistical significance was assessed with chi-square tests for categorical variables and t-tests for continuous variables. BMI-SDS: body mass index. SD: Standard deviation.

* P<0.05.

**Baseline anthropometrics in parents:** The number of overweight or obese mothers and fathers did not differ between participating and non-participating families. The only significant association was found between father's weight status and participation, with fathers of participating children having a higher BMI than fathers of non-participating children (Table 2).

**Table 2:** Baseline characteristics of parents of participating and nonparticipating children.

Variable	Participants (n = 110)	Nonparticipants (n = 100)	P-value
**Anthropometric measurement**			
Mother's age (mean ±SD)	39.2±6.2	38.5±5.5	P = 0.436
Father's age (mean ± SD)	42.1±6.4	41.3±6.6	P = 0.436
Mother's BMI	28.0±5.9	27.7±6.5	P = 0.74
Father's BMI	28.5±4.5	27.2±3.7	P = 0.04*
**Socio-demographic**			
Mother's education			P = 0.819
*Low*	7.3%	8.0%	
*Middle*	66.1%	70.0%	
*High*	26.6%	22.0%	
Father's education			P = 0.636
*Low*	15.9%	15.7%	
*Middle*	60.4%	62.7%	
*High*	23.8%	21.6%	
Mother's employment			P = 0.935
*No employment*	22.9%	22.0%	
*Working part-time*	46.7%	45.0%	
*Working full-time*	30.3%	33.0%	
Father's employment			P = 0.276
*No employment*	11.9%	9.9%	
*Working part-time*	1.0%	4.9%	
*Working full-time*	87.1%	85.2%	
Household-net-income			P<0.001*
*<2000 €*	38.1%	45.4%	
*2000–4000 €*	55.3%	52.,6%	
*>4000 €*	6.7%	2.0%	
Size of the hometown			P = 0.049*
*Village*	37.7%	40.0%	
*Small town*	21.1%	12.0%	
*Mid-size town*	11.0%	25.0%	
*Big city*	30.3%	23.0%	
Marital status			P = 0.189
*Married or living together*	78.2%	67.0%	
*Remarried or living together with new partner*	7.2%	12.0%	
*Living alone (widowed, single, divorced)*	14.5%	21.0%	
**Behavioral variables**			P = 0.050*
*Stages of behavior change*			
Motivation phase			
*Non-intention*	3.1%	6.8%	
*Intention*	19.4%	18.2%	
Planning phase	20.4%	14.8%	
Action phase	40.8%	35.2%	
Maintenance	16.3%	25.0%	

Statistical significance was assessed with testing for categorical variables and t-tests for continuous variables.

* P<0.05.

**Gender differences between participating and non-participating families:** Families with girls participated more often in the intervention than families with boys (p = 0.008), and the participating girls were significantly younger in age than participating boys (t[239] = 3.97, p<0.001) (Table 1).

**Eating behavior, physical activity and leisure time:** There was a strong relationship between the regularity of breakfast consumption as well as meal frequency of children and adolescents and program participation: non-participating children had irregular and infrequent breakfasts, corresponding to the observed decrease in meal frequency among non-participating children (Table 1). Levels of physical activity, leisure time habits and media consumption in children and adolescents did not differ significantly between both groups (Table 1). With regard to parents, meal pattern, physical activity, leisure-time habits and media consumption did not differ between parents of participating and non-participating children (Table 2).

**Subjective well-being and quality of life in children and adolescents:** A significant relationship between the quality of life and study participation was observed: Non-participating children had better subjective physical well-being than participating children (p = 0.018). Other dimensions of health-related quality of life (emotional well-being, self-esteem, family, friends, and everyday functioning) did not differ significantly between participating and non-participating children.

**Parental change in lifestyle:** The greatest motivational difference between parents of participating and non-participating children was the willingness to change their lifestyle: parents of nonparticipants believed that they can easily maintain a healthy lifestyle. In contrast, parents of participants demonstrated difficulties in planning or preparing for a change in lifestyle, or actually engaging in efforts to change the lifestyle (X^2^[1, N = 186] = 3.834, p = 0.05).

**Socio-demographic characteristics between participating and non-participating families:** Household-net-income was significantly lower in nonparticipants than participants (X^2^[2, N = 202] = 3.106, p<0.001). No significant association was observed regarding participation and marital status, parental employment, parental level of education, or ethnicity. (Table 2).

### Non-modifiable reasons for nonparticipation

Out of the 227 families who denied program participation but agreed to complete identical questionnaires than the intervention group for analysis of reasons for (non)participation, 35 families had to be excluded due to non-modifiable reasons for nonparticipation. These non-modifiable exclusion criteria included chronic underlying medical illness of the child (n = 3), BMI-reduction into the normal weight range of a respective child between screening and enrolment (n = 6), and the child not living with its parents but within a children's local residential community (n = 1). 25 families had to be excluded because they sent their written consent for study participation to the study centre, but never returned the completed questionnaires.

### Subjectively stated reasons for nonparticipation

**Figure 1:**
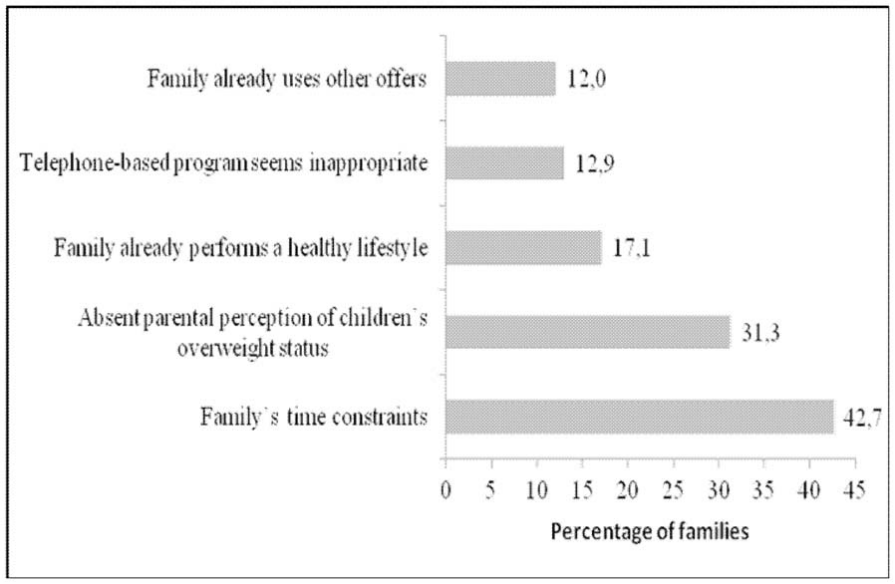
Main reasons for nonparticipation given by parents from a defined list. Multiple answers were possible.

Nonparticipants stated that organizational issues (i.e. time constraints), and non-perception of a child's overweight played an important role in their decision to decline participation. Participation in other programs and using other offers were also perceived as an important reason for nonparticipation. Financial arguments, as well as inconvenient timing due to critical life events were additional factors associated to nonparticipation in the program. The main reason for nonparticipation was the belief to already practice a healthy lifestyle (figure 1).

### Factors related to program participation

Binary logistic regression was performed to investigate the factors associated with participation including children's BMI-SDS, gender of the child, physical activity, physical well-being, meal frequency, household-net-income, and place of residence. The variance explained by the model selected was 24.0% (R^2^). The model was able to correctly classify 72.3% of those subjects who participated in the program, and 65.2% of those who did not, for an overall success rate of 68.5%. Table 3 presents the remaining effects of those variables to program participation. Children's weight was related to participation, corresponding to the univariate analyses. In accordance, lower BMI-SDS of the child prior to enrolment, lower meal frequency, residence in a mid-size town, low household-net-income and superior physical well-being of children were related to nonparticipation.

**Table 3:** Factors associated with program participation.

Predictors in the Regression	B	S.E.	Wald	Df	P	Odds Ratio
BMI-SDS	0.949	0.337	7.958	1	= 0.005	2.584
Meal frequency	−0.840	0.235	12.803	1	<0.001	0.432
Physical well-being	0.023	0.009	7.036	1	<00.05	1.023
Household-net-income	−0.316	0.180	3.086	1	= 0.079	0.729
Place of residence			10.323	4	<00.05	

B = unstandardized coefficient.

Excluded variables: physical activity, gender.

## Discussion

The identification of reasons for nonparticipation in obesity intervention programs is essential, since response rates remain very low. Family-based lifestyle interventions with a behavioral program aimed at sustainably changing the family's lifestyle (“thinking patterns”) have been shown to result in significant and clinically meaningful decrease in childhood overweight [9], and programs which mainly address parents and train parenting skills on healthy lifestyle have been shown to achieve significant weight loss in younger children [22]. Parenting styles and the involvement of the family environment have consistently been identified as crucial factors linked to childhood obesity and the success of intervention strategies [23]–[25]. However, motivating families to participate in prevention programs has been difficult, particularly for family-based interventions [26]. Therefore, reasons for participation and nonparticipation in our program have been analyzed in the present study. We provide evidence that potential nonparticipants may not perceive a need for action: The weight status of the children of eligible families was clearly the most obvious reason for nonparticipation, as the majority of children enrolled in our program were already obese, whereas families who denied program participation had significantly more often “only” overweight children. These results demonstrate that poor self-perception of the children's weight status was a common reason for parents to deny program participation. This is consistent with another study showing that 50% of the parents do not recognize that their child is overweight and that parents of obese children show greater awareness of the children's weight status than parents of overweight children [14]. Treatment motivation is much lower in families with overweight children compared to families with obese children [13]. Parents, in general, tend to underestimate their children's weight status, although being overweight is recognized as a health problem [27]–[29], and parent's readiness to change life style habits depends on whether they perceive themselves to be overweight [30].

More families with girls were willing to participate in our program, and participating girls were younger than participating boys. It might be speculated that due to the actual “image of perfect beauty” particularly families with overweight or obese girls feel more addressed by obesity prevention programs than families with obese boys. The phenomenon of gender differences in the effectiveness of intervention programs is well known [31]–[32].

Parents control many aspects of the nutrition and physical environment that contribute to a child's health-related behavior [33]–[34], and parents identify many barriers that their children face in adopting prevention recommendations [35]–[36]. In our study, the biggest motivational difference between participants and nonparticipants was the willingness to change their lifestyle. Parents of non-participating children had the conviction that their children already perform a healthy lifestyle. However, in contrast to the subjective belief of living healthy, we show that non-participating families have unbalanced meal patterns (i.e. irregular and infrequent breakfasts and lower meal frequency). High meal frequency has been shown to be inversely associated with childhood obesity, and skipping breakfasts is significantly related to a higher prevalence of childhood obesity [37]. An additional factor that we show to be significantly associated with program participation is quality of life: A poor quality of life in overweight/obese children and adolescents (especially physical well-being) is a prerequisite for their acceptance of need for action and program participation. This is in accordance with previous studies [38]–[39].

Children of parents with low socio-demographic status are more likely to be overweight or obese, and there is a strong relationship between single-parent status and excess weight in children [40]. Our results indicate that sociodemographic status only partially affected an individual's willingness to participate in our obesity intervention program. There were no differences between participating and non-participating families in terms of marital status, parental education or employment. The only difference was a significantly lower household-net-income in nonparticipants. In addition, financial investments were experienced as a barrier in this group. Thus, parental and family characteristics rarely contributed to participation in our program. This is consistent with another study which failed to identify various family factors as significant predictors of participation in an obesity intervention program [41]. The main reason for nonparticipation in our program, as determined by open questions, was time constraints of the family, followed by an absent parental perception of children's overweight status and the believe that the family already performs a healthy lifestyle. Strictly telephone-based interventions are currently rare. To the best of our knowledge, no other study has to date offered telephone counseling to families with overweight or obese children. However, there is solid evidence that telephone counseling interventions may significantly and successfully change lifestyle behavior in adults, but the acceptance of new media in obesity prevention is low and should be promoted [42]. Thus, the work presented herein might be of importance for the development of future prevention programs addressing family based settings.

As many obese children remain obese adults and develop metabolic and/or cardiovascular co-morbidities later in life [5]–[6], it is not only crucial to develop validated and evidence-based prevention programs for childhood obesity, but also to develop effective programs to prevent features of the metabolic syndrome in adulthood. It is well established that diabetes mellitus is preventable with lifestyle intervention and that moderate changes in diet and physical activity may reduce the incidence of type 2 diabetes mellitus (T2DM) in individuals with impaired glucose tolerance [43]. Thus, it has been suggested that research should now focus on developing efficient screening and risk-identification strategies and realistic scenarios for public-health policy to implement validated prevention programs into daily practice [43]. In order to standardize interventions for diabetes prevention programs for adults in Europe, a guideline has been established with the aims to a) identify candidates with increased risk to develop type 2 diabetes mellitus and to b) guide identified patients through the initiation and management of an appropriate lifestyle intervention to prevent type 2 diabetes [44]–[45]. However, identification of participation barriers is an important prerequisite towards the development of prevention programs [43].

Our study has several strengths. First, to the best of our knowledge, no previous study has investigated reasons for nonparticipation in an obesity prevention programs in such a large cohort (n = 433; 241 participants and 192 nonparticipants), including such a wide age span of childhood development (3.5–17.4 years). Second, comprehensive and identical baseline information was obtained from participants and nonparticipants, including anthropometric and clinical data, lifestyle, eating patterns, sociodemographic and socioeconomic parameters, quality of life. Third, all information was obtained separately from the family triad of mother, father, and child (older than 10 years). We are not aware of any study that has obtained information from both the mother and father of the family within the same setting. However, this study also has certain limitations. First, the study was limited to a cohort of families recruited to be overweight or obese, but refused to participate in a telephone-based prevention program, which may have led to selection bias. Second, the multivariate binary logistic regression model could explain only 24% of the variance, indicating that variables that were not included in the model may contribute to family participation or nonparticipation, respectively, in obesity prevention programs. Future research for programs targeted to prevent childhood obesity should focus on reasons for - not only against - family participation.

### Conclusion

The characterization of reasons for participation or nonparticipation in obesity prevention programs is crucial for the development of effective programs to address childhood obesity. The family setting has been identified as a potential “delivery system” for such programs; however, we show that a large number of families with overweight children do not feel addressed, and that the majority of participating families have children who are already obese. Non-participants, who were often low-income families, believed that they already perform a healthy lifestyle and had a higher subjective wellbeing. Thus, even a low-threshold obesity prevention program does not reach the families who really need it.
